# CD169+ Monocyte and Regulatory T Cell Subsets Are Associated with Disease Activity in Rheumatoid Arthritis

**DOI:** 10.3390/jpm12111875

**Published:** 2022-11-09

**Authors:** Amanda J. Eakin, Tahanver Ahmed, Cathy M. McGeough, Stephen Drain, H. Denis Alexander, Gary D. Wright, Philip V. Gardiner, Dawn Small, Anthony J. Bjourson, David S. Gibson

**Affiliations:** 1 Personalised Medicine Centre, School of Medicine, Ulster University, Altnagelvin Hospital, Londonderry BT47 6SB, UK; 2 The Patrick G Johnston Centre for Cancer Research, Queen’s University Belfast, Belfast BT9 7AE, UK; 3 Biomarker Research Unit, Almac Diagnostics Ltd., Craigavon BT63 5QD, UK; 4 Department of Rheumatology, Musgrave Park Hospital, Belfast BT9 7JB, UK; 5 Department of Rheumatology, Altnagelvin Hospital, Londonderry BT47 6LS, UK

**Keywords:** monocyte, regulatory T cell, cytokine, inflammation, rheumatoid arthritis

## Abstract

Disease activity in rheumatoid arthritis (RA) is influenced by activation of circulating and synovial immune cells. Regulatory T cells (Tregs) and monocytes are key cells that drive inflammation in RA. This study investigated if a relationship exists between disease activity in RA and circulating Treg and monocyte numbers and phenotypes. A potential sialic acid (Sia) mediated link between Tregs and monocytes was also probed in vitro. Peripheral blood mononuclear cells (PBMCs) were isolated from RA patient (*n* = 62) and healthy control (*n* = 21) blood using density gradient separation. Flow cytometry was used to count and phenotype Treg and monocyte subsets, and to sort healthy control Tregs for Sia cell culture experiments. The effects of Sia on activated Treg FoxP3 and NFκB expression was assessed by flow cytometry and concentrations of secreted TNFα, IL-10 and IFNγ determined by ELISA. High disease activity RA patients who were unresponsive to disease modifying anti-rheumatic drugs (*n* = 31), have significantly lower relative numbers (percentages) of CD4^+^CD25^+^CD127^−^ Tregs (*p* < 0.01) and memory CD45RA^−^FoxP3^+^ Tregs (*p* < 0.01), compared to low disease activity responders (*n* = 24). Relative numbers of non-classical CD169^+^ monocytes are associated with disease activity in RA (*p* = 0.012). Sia reduced Treg expression of FoxP3, NFκB and cytokines in vitro. A strong association has been identified between non-classical CD169^+^ monocytes and post-treatment disease activity in RA. This study also indicates that Sia can reduce Treg activation and cytokine release. We postulate that such a reduction could be mediated by interaction with sialyted proteins captured by CD169^+^ monocytes.

## 1. Introduction

Rheumatoid arthritis (RA) is a chronic condition characterised by an autoimmune response targeted primarily at synovial joints. Tenderness and swelling of joints are typical symptoms of the disease, which is also characterised by elevated blood-based inflammatory markers C-reactive protein (CRP) and erythrocyte sedimentation rate (ESR). These outcome measures are combined in a composite Disease Activity Score of 28 joints (DAS28), measurement/level along with the patient’s rating of their general health on a 0–10 scale. The DAS28 allows clinicians to monitor disease activity and assists in determining therapeutic responses. However, composite disease activity scores such as the DAS28 may be unreliable. The ESR and CRP components of DAS28 are non-specific markers of RA disease activity [1,2,3]. The visual analogue scale (VAS) and tender joint count components of DAS28 can also be subjective, further emphasizing the need for more accurate and reliable measures of disease activity.

Disease activity in RA stems from activation of immune cells found in peripheral blood (PB) and affected synovial tissue [4]. Once activated, these immune cells can directly or indirectly promote secretion of degradative enzymes within the synovial joint, increasing cartilage breakdown and bone erosion [5,6]. T cells play a pivotal role in moderating the adaptive immune response in healthy individuals [7]. Specifically, regulatory T cells (Tregs) reduce the actions of damage-causing effector T cells (Teffs). However, in the RA autoimmune environs, Treg suppression of Teffs is less effective. Therefore, secretion of cytokines and recruitment of other immune cells to the synovial joint by Teffs is unchecked, thus increasing the risk of irreversible damage [8,9,10].

CD4^+^CD25^+^ Treg density is increased in the synovial fluid of RA patients compared to PB, and overall Treg numbers are elevated in RA compared to healthy indivduals [8,10]. Furthermore, synovial fluid Tregs exhibit elevated activation markers including transcription factor Forkhead box P3 (FoxP3) and cytotoxic T lymphocyte protein-4 (CTLA-4) [8,10]. A recent study observed increased CD4^+^CD25^+^FoxP3^+^ Treg numbers with lower expression of activation markers CD69 and CD71 in RA patients [11]. However, the association with individual responses to treatment were unclear. When patients were classified according to anti-TNF response, increased numbers of CD4^+^CD25^+^ Tregs were observed in PB of responders relative to non-responders [9]. This finding was also associated with reduced CRP levels in responders, suggesting peripheral Treg numbers may be associated with disease activity and drug response in RA. However, further evidence is needed to clearly define changes in relative numbers of peripheral Tregs, their specific phenotypes and activation levels in both treatment response and non-response.

Treg activity during inflammatory episodes is influenced by interaction with monocytes and macrophages [12]. In vitro studies have demonstrated the ability of synovial monocytes to promote Th1 and Th17 responses in CD4^+^ T cells [13]. For example, intracellular levels of IL-17, IFN-γ, TNFα and IL-10 increase in Tregs as a result of exposure to activated monocytes [14]. Furthermore, increased IL-6 and IL-23 secretion from monocyte derived dendritic cells, has been associated with the induction of Th17 cells and Tregs in RA [15].

Monocyte phenotypes may also represent surrogate markers of disease activity in RA. Sialic acid-binding immunoglobulin-like lectin 1 (CD169, also known as Siglec-1), which is an adhesion molecule restricted to monocytes and macrophages, is elevated both intracellularly and at the cell surface in line with increases in DAS28 and CRP [16,17]. Furthermore, CD169 levels subside along with disease activity improvements in response to treatment [17]. The mechanism behind the CD169 elevation in RA is not fully understood, however it may be implicated in pro-inflammatory pathways, resulting in interferon-gamma (IFN-ɣ) secretion from activated T cells [18,19]. Furthermore, CD169 binds to ligands on the surface of Tregs, an action that may contribute to Treg modulation in RA [20]. CD43, a cell-surface sialoglycoprotein, is one such Treg based CD169 ligand that has previously been associated with in vitro Treg activation [21,22]. However, a study of the interaction between Tregs and monocytes via CD43 and CD169 has not yet been made.

Xiong et al. describe increased CD169 expression on CD14^+^CD16^+^ resident monocytes as well as CD14^+^CD16^−^ classical monocytes of RA patients, compared to healthy controls [17]. Resident monocytes can be further subdivided into CD14^high^CD16^+^ intermediate and CD14^low^CD16^+^ non-classical monocytes [23,24]. A number of studies highlight the importance of considering these three monocyte subsets in RA. Synovial CD16^+^ monocytes increase in RA [25,26], whereas PB CD16^−^ monocytes are reduced in RA compared to healthy controls [27]. Furthermore, several studies showcase the potential for baseline monocyte subsets to predict treatment responses in RA to anti-TNF therapy and methotrexate (MTX) [28,29], where frequencies of classical and intermediate monocytes increase in non-responders compared to responders [28]. However, further evidence is needed to fully understand the balance between monocytes subsets and disease activity measures in RA, specifically in relation to CD169^+^ subpopulations.

Therefore, the primary aim of this study was to investigate if associations exist between Treg activation and disease activity or conventional disease modifying anti-rheumatic drug (DMARD) response in RA. The secondary aims of the study were to investigate if CD169^+^ monocyte subsets are associated with disease activity and examine if sialic acid carried by monocytes modulates Treg activity in vitro.

## 2. Materials and Methods

### 2.1. Study Design and Participants

Informed consent was obtained from all patients and healthy control subjects. The research team at Ulster University collaborated with rheumatologists from the Western Health and Social Care Trust and the Belfast Health and Social Care Trust to design, conduct and recruit patients to the study. All patients fulfilled the 2010 American College of Rheumatology (ACR) criteria for Rheumatoid Arthritis [30]. Patients were treated with one or more conventional disease-modifying anti-rheumatic drugs (DMARD), including MTX, sulfasalazine, leflunomide and hydroxychloroquine. Patients were classified as ‘responders’ or ‘non-responders’ according to the National Institute for Health and Clinical Excellence (NICE) guidelines, where responders exhibit a change in DAS28-ESR of >1.2 following treatment [31]. Blood samples and retrospective clinical data were collected at the time of consent (Table 1). Healthy control subjects were also recruited, excluding individuals with chronic inflammatory or autoimmune disorders.

### 2.2. Blood Processing

Blood samples collected for PB mononuclear cell (PBMC) isolation were collected in tripotassium ethylenediaminetetraacetic acid (K3EDTA) coated tubes (Aquilant Scientific, UK). Phlebotomy was performed by a research nurse or by a qualified member of the research team. Blood tubes were stored at room temperature until processing. Whole blood was diluted 1:1 in BD Cell Wash (Becton Dickinson) prior to PBMC isolation. Histopaque-1077 (Sigma Aldrich, Gillingham, UK) density-gradient buffer was used to isolate PBMCs from blood samples, whereby the diluted blood was layered into the buffer according to the manufacturer’s instructions. The tube was centrifuged at 400 rcf for 30 min at 18 °C. After centrifugation, the majority of the upper plasma layer was discarded, leaving approximately 0.5 mL above the PBMC layer. The PBMC layer was dispensed into a clean tube along with the remaining plasma layer and approximately half of the Histopaque layer. BD Cell wash (Becton Dickinson) was added to the crude extracted PBMCs, increasing the total volume in the tube to at least 3 times the original volume. The tube was inverted 3–4 times to ensure sufficient mixing and then was centrifuged at 300 rcf for 10 min at 18 °C. The supernatant was discarded and the cell pellet was washed again a further 2 times in the same volume of BD Cell wash (Becton Dickinson). After final centrifugation, the supernatant was discarded and the cell pellet was resuspended in the required volume of BD Cell wash (Becton Dickinson) for staining.

### 2.3. Cell Immunophenotyping

PBMCs were labelled with antibodies to CD3, CD4, CD25, CD45, CD45RA, CD127 and CD43, according to the manufacturer’s instructions to assess relative Treg numbers (results are expressed as relative frequency [percentages] and/or absolute number [×10^9^cells/L]) (Becton Dickinson, Wokingham, UK). This study used a CD4^+^CD25^+^CD127^−^ phenotype to define Tregs according to previous literature [32]. When FoxP3 and nuclear factor kappa-light-chain-enhancer of activated B cells (NF-κB) antibody labelling was required, cells were pretreated with Permeabilising solution 2 (Catalogue number 340973, Becton Dickinson, UK) in order to stain these intracellular components. For monocyte analysis, cells were firstly enriched using a pan monocyte isolation kit and the autoMACS Pro separator (Miltenyi Biotec) as this improved CD169 staining downstream of enrichment as tested using flow cytometry. Enriched monocytes were labelled with antibodies to CD14, CD16 and CD169 (results are expressed as relative frequency [percentages] and/or absolute number [×10^9^cells/L]) (Becton Dickinson, UK). Cell populations were analysed using a fluorescence-activated cell sorter (FACS) Aria III with BD FACSDiva software (version 8.0.1) (Becton Dickinson, UK). Negative controls including unlabelled cells and isotype matched control antibodies are used to optimise gating strategies. The positivity of a specific cell surface marker in labelled samples compared to the negative control was analysed as a percentage of the parent population. The median fluorescence intensity (MFI), indicating cell surface density of a specific marker, was also noted for cell populations.

### 2.4. Cell Expansion and Culture

CD4^+^CD25^+^CD127^−^ Treg cells were sorted by FACS into PBS containing 20% AB serum (Sigma Aldrich, UK), then washed and resuspended in TexMACS^TM^ medium (Miltenyi Biotec, Woking, UK), supplemented with 5% AB serum (Sigma Aldrich), 1% Penicillin-Streptomycin (Gibco, Ireland) and human recombinant IL-2 (Miltenyi Biotec, UK). Trypan blue (Sigma Aldrich, UK) was used to ensure cell viability after sorting was >90–95%. The Miltenyi Biotec human Treg expansion kit induced Treg proliferation, expanding the population to sufficient cell numbers for experiments. Following 14 days of expansion, Tregs were stimulated with 10 ng/mL phorbol 12-myristate 13-acetate (PMA) (Sigma Aldrich, UK) and 500 ng/mL ionomycin (IO) (Invitrogen) for 24 h. Prior to stimulation, PMA was reconstituted in 99.8% ethanol (Sigma Aldrich) to make a stock solution of 1 mg/mL. The stock solution was diluted as required using media. IO was reconstituted in deionized distilled water (ddH_2_O) to make a stock solution of 1 mg/mL. The stock solution was further diluted to 500 ng/mL using media. PMA stimulates a nuclear factor-kappa B (NF-κB) activated T cell signature in vitro via protein kinase C [33]. During the same 24-h stimulation, Tregs were incubated with or without 10 mM sialic acid (Sia) (Sigma Aldrich, UK). Monomeric Sia was reconstituted in ddH_2_O to make a stock solution of 50 mg/mL (Sigma Aldrich). Then, 10 mM of Sia was added directly to each well as required from the stock solution. In the case of each stimulant, a vehicle control was assessed to ensure no effect was observed from the diluent alone. At baseline, 24 h and 48 h time points (Day 0, Day 1 and Day 2), cells were removed from culture and labelled for FACS analysis of extracellular markers CD4, CD25, CD127 and CD43, and intracellular markers NFκB and FoxP3. Cell culture conditioned media was retained and secreted cytokine concentrations determined by ELISA, below. A preliminary experiment included a Day 2 time-point, however latter experiments were concluded at the Day 1 time-point as this was sufficient to observe Sia treatment effects. The experiment had a total of 2 biological replicates, each having 2 technical replicates.

### 2.5. Enzyme-Linked Immunosorbent Assay (ELISA)

Conditioned media were analysed by DuoSet ELISA to quantify TNFα (Cat. Number DY210), IL-10 (Cat. Number DY217B) and IFN-γ (Cat. Number DY285B) according to the manufacturer’s instructions (R&D Systems, Abingdon, UK). The absorbance at 450 nm was determined using an Epoch plate reader (Biotek Ltd., Ashcott, UK) with Gen5^TM^ software, with wavelength correction at 540 nm. The cytokine concentrations within the conditioned media were interpolated from the constructed standard curve.

### 2.6. Statistical Analysis

Mann–Whitney tests were used to assess the statistical significance of differences between study subgroups, as the comparisons are between ‘independent’ sample groups. Furthermore, when flow cytometry data was plotted in SPSS (Data analysis software), it was not normally distributed and therefore a Mann–Whitney test was applied. Unpaired t-tests were used to analyse the significance of differences in ELISA data. Graphs shown depict median values with error bars of interquartile range. Statistical analysis was performed with GraphPad Prism (version 5.01).

## 3. Results

### 3.1. Study Participant Demographics

Participant demographics were summarised for DMARD responder and non-responder subgroups, as median ± standard deviation (SD) (Table 2). The median disease duration and the median DAS28-ESR scores were significantly higher in non-responder patients (9.00 years; 5.51 DAS28) compared to responders (4.50 years; 2.34 DAS28) (*p* < 0.01). Median ESR was also significantly increased in non-responders (21.5 mm/h) compared to responders (11.0 mm/h) (*p* = 0.01), however there was no statistically significant difference between CRP levels of each group (*p* = 0.08).

### 3.2. Frequency of Circulating Tregs in RA

**Figure 1:**
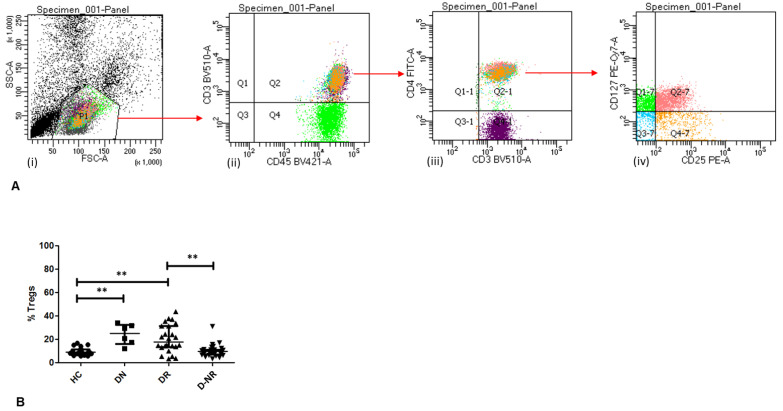
(**A**) FACS dot plot showing gating of (**i**) lymphocyte population and subsequent CD3^+^CD45^+^CD4^+^CD25^+^CD127^−^ Tregs ((**ii**)–(**iv**)) of a typical RA patient sample. (**B**) The frequencies CD4^+^CD25^+^CD127^−^ Tregs in healthy controls and RA patients were calculated as a % of CD3^+^CD45^+^ T cells. *p* values shown were obtained using Mann–Whitney tests; **, *p* < 0.01. Central bar represents median value; error bars represent interquartile range. HC = healthy controls (*n* = 21), DN = DMARD naïve (*n* = 6), DR = DMARD responders (*n* = 24) and D-NR = DMARD non-responders (*n* = 31).

CD4^+^CD25^+^CD127^−^ Treg frequencies from healthy subjects and RA patient subgroups were calculated as a percentage of CD3^+^CD45^+^ T cells (Figure 1). Circulating relative Treg frequencies were significantly increased in DMARD naïve (25.00 ± 8.77, *n* = 6, *p* < 0.01) and DMARD responder RA patients (17.80 ± 11.75, *n* = 24, *p* < 0.01) compared to healthy controls (9.10 ± 3.21, *n* = 21), although the number of DMARD naïve patients is relatively low. Furthermore, RA patients who were unresponsive to DMARD treatment had a significantly lower percentage of circulating Tregs (9.50 ± 4.98, *n* = 31, *p* < 0.01) compared to those who have responded.

### 3.3. Treg Activation in RA (FoxP3 and CD45RA)

**Figure 2:**
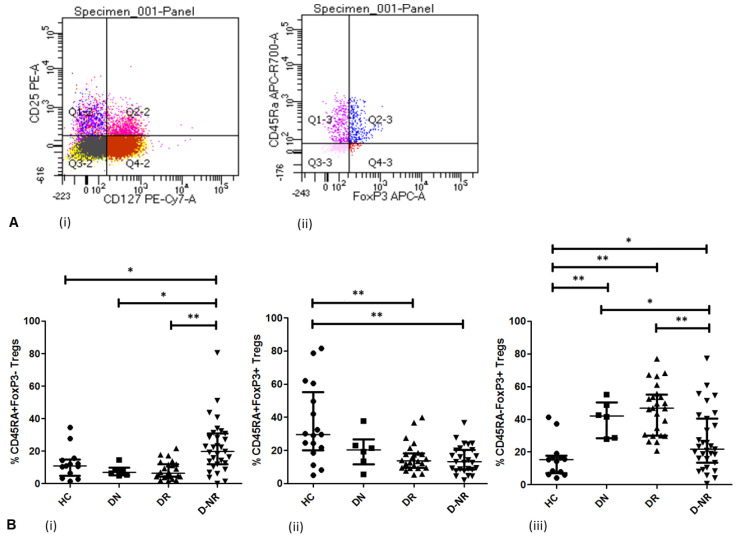
(**A**) Representative flow cytometry dot plot and gating strategy of (**ii**) FoxP3^+/−^ and CD45RA^+/−^ Tregs following (**i**) CD4^+^CD25^+^CD127^−^ Treg gating. (**B**) Frequencies of naïve (**i**) CD45RA^+^FoxP3^−^ and (**ii**) CD45RA^+^FoxP3^+^ subsets as well as (**iii**) memory CD45RA^−^FoxP3^+^ cells were analysed in circulating Tregs of RA patients and healthy controls and calculated as a % of CD3^+^CD25^+^CD127^−^ Tregs. *p* values were calculated using Mann–Whitney tests; *, *p* < 0.05; **, *p* < 0.01. Central bar represents median value, error bars represent interquartile range. HC = healthy controls (*n* = 13), DN = DMARD naïve (*n* = 6), DR = DMARD responder (*n* = 24), D-NR = DMARD non-responder (*n* = 31).

The relative frequencies of CD45RA^+^ and activated FoxP3^+^ Tregs (CD4^+^CD25^+^CD127^−^) were analysed in healthy controls and RA patients (Figure 2). The frequency of CD45RA^+^FoxP3^−^ Tregs was significantly higher in DMARD non-responders (19.90% ± 16.39, *n* = 31), compared to healthy controls (10.90% ± 9.47, *n* = 13, *p* < 0.05), DMARD naïve (6.90% ± 3.51, *n* = 6, *p* < 0.05) and DMARD responder patients (6.50% ± 5.54, *n* = 24, *p* < 0.01) (Figure 2A). Furthermore, the frequency of CD45RA^+^FoxP3^+^ Tregs was significantly reduced in DMARD non-responders (13.20% ± 8.33, *n* = 27) and DMARD responders (13.60% ± 8.81, *n* = 24, *p* < 0.01) compared to healthy controls (29.60% ± 23.27, *n* = 13, *p* < 0.01) (Figure 2B). The frequency of CD45RA^−^FoxP3^+^ Tregs was significantly elevated in DMARD naïve (42.00% ± 10.82, *n* = 6, *p* < 0.01), DMARD responder (46.70% ± 15.42, *n* = 24, *p* < 0.01) and DMARD non-responder patients (21.60% ± 18.66, *n* = 31, *p* < 0.05), in comparison to healthy controls (15.40% ± 11.44, *n* = 13), although the number of DMARD naïve patients was relatively low (Figure 2C). However, DMARD non-responders exhibited a significantly lower frequency of CD45RA^−^FoxP3^+^ Tregs compared to DMARD naïve (*p* < 0.05) and DMARD responder patients (*p* < 0.01).

### 3.4. Monocytes in Health and RA Subgroups

**Figure 3:**
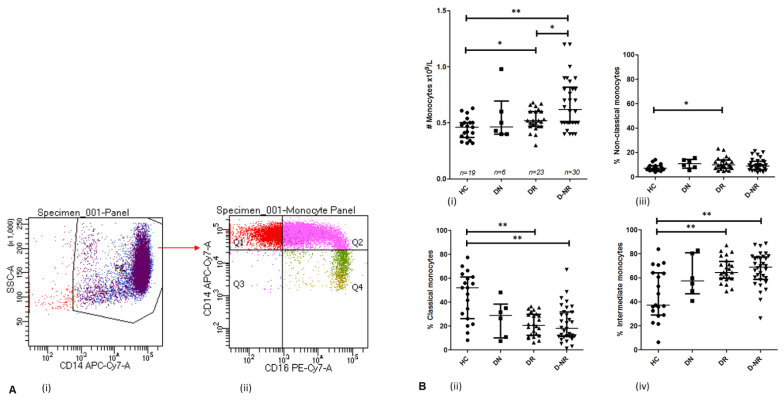
(**A**) Representative FACS dot plots and gating strategy of enriched monocytes, whereby gating position was determined by negative controls. (**i**) CD14^+^ monocytes were initially gated. (**ii**) Monocyte subsets were subdivided into CD14^++^CD16^−^ classical (Q1), CD14^++^CD16^+^ intermediate (Q2) and CD14^+^CD16^++^ non-classical (Q4). (**B**) (**i**) The absolute number of monocytes as measured by full blood count (FBC) in healthy controls and RA patients. The frequency of classical (**ii**), non-classical (**iii**) and intermediate (**iv**) monocytes in healthy controls and RA patients were calculated as a % of CD14^+^ monocytes. *p* values shown were obtained using unpaired *t*-tests or Mann–Whitney tests depending on normality of distribution; *, *p* < 0.05; **, *p* < 0.01. Central bar represents median value, error bars represent interquartile range. HC = healthy controls, DN = DMARD naïve, DR = DMARD responder, D-NR = DMARD non-responder.

The absolute number of circulating monocytes in DMARD responders (0.527 × 10^9^/L ± 0.097, *n* = 23) and non-responders (0.670 × 10^9^/L ± 0.229, *n* = 30) are significantly higher relative to healthy controls (0.452 × 10^9^/L ± 0.097, *n* = 19, *p* = 0.019 and 0.0003 respectively) (Figure 3A). There is no statistically significant difference between absolute monocyte numbers in healthy controls and DMARD naïve patients (0.552 × 10^9^/L ± 0.223, *n* = 6, *p* = 0.407). Additionally, the absolute number of monocytes in responders is significantly lower than non-responders (*p* = 0.041).

Monocyte subsets were assessed as shown in Figure 3. The frequency of CD14^++^CD16^−^ classical monocytes is significantly lower in RA responders (21.37% ± 9.68, *n* = 24) and DMARD non-responders (22.09% ± 15.03, *n* = 32), compared to healthy controls (45.03% ± 20.33, *n* = 19, *p* < 0.001) (Figure 3B). However, the frequency of CD14^+^CD16^++^ non-classical monocytes is increased in RA patients compared to healthy controls (7.595% ± 2.606, *n* = 19), the difference statistically significant in DMARD responders (10.93% ± 4.978, *n* = 24, *p* = 0.017) (Figure 3C). Furthermore, the frequency of CD14^++^CD16^+^ intermediate monocytes is significantly increased in both responders (66.37% ± 10.31, *n* = 24, *p* = 0.004) and non-responders (66.90% ± 13.90, *n* = 32, *p* = 0.001) compared to healthy controls (45.83% ± 21.31, *n* = 19) (Figure 3D). No statistically significant difference is observed between the frequencies of monocyte subsets of responders and non-responders.

### 3.5. CD169 Expression on Monocyte Subsets in RA and Health

**Figure 4:**
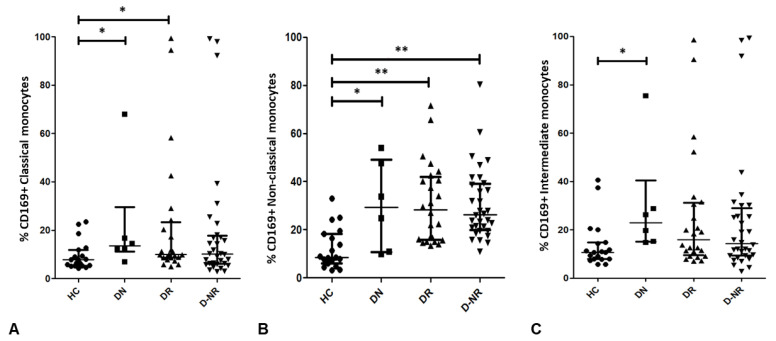
The frequency of CD169^+^ classical (**A**), non-classical (**B**) and intermediate (**C**) monocytes in healthy controls and RA patients were calculated as a % of CD14^+^ monocytes. *p* values shown were calculated using Mann–Whitney tests; *, *p* < 0.05; **, *p* < 0.01. Error bars represent interquartile range. HC = healthy controls (*n* = 19), DN = DMARD naïve (*n* = 6), DR = DMARD responder (*n* = 24), D-NR = DMARD non-responder (*n* = 32).

Overall, the percentage of CD169 positive monocytes is elevated in RA patient subgroups compared to healthy controls (Figure 4). DMARD naïve RA patients (21.83% ± 22.80, *n* = 6, *p* = 0.039) and DMARD responders (21.89% ± 26.31, *n* = 24, *p* = 0.014) have a significantly increased relative number of CD169+ classical monocytes, compared to healthy controls (9.426% ± 5.890, *n* = 19), although DMARD naïve patient number is relatively low. However, the difference was not statistically significant in DMARD non-responders (19.83% ± 26.40, *n* = 32, *p* = 0.185) compared to healthy controls (Figure 4A). Similarly in non-classical monocytes, CD169 percentage is increased in DMARD naïve (30.10% ± 18.41, 0.014), DMARD responder (30.95% ± 16.73, *p* < 0.001) and DMARD non-responder (30.68% ± 15.05, *p* < 0.001) RA patients compared to healthy controls (12.15% ± 8.392) (Figure 4B). Additionally, CD169 percentage is increased in intermediate monocytes for each patient group compared to healthy controls (13.87% ± 9.778), however only with statistical significance in DMARD naïve patients (30.02% ± 23.00, *p* = 0.011) (Figure 4C). Furthermore, the MFI of CD169 on the surface of classical and intermediate monocytes is not significantly increased in DMARD non-responders compared to other patient groups and healthy controls.

### 3.6. Relationship between CD169^+^ Monocytes or CD43^+^ Tregs and Disease Activity

**Figure 5:**
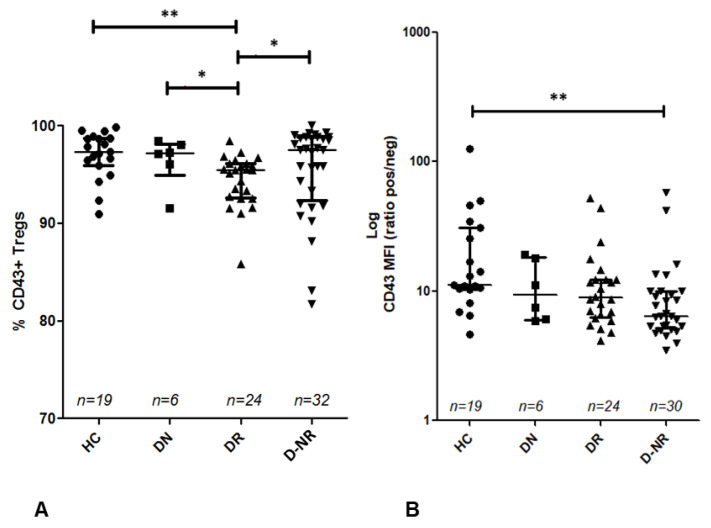
(**A**) The frequency of CD43^+^ Tregs in healthy controls and RA patients were calculated as a % of CD4^+^CD25^+^CD127^−^ cells. (**B**) A log scale graph of CD43 MFI in Tregs of healthy controls and RA patients. *p* values shown were calculated using Mann–Whitney tests; *, *p* < 0.05; **, *p* < 0.01. Central bar represents median value, error bars represent interquartile range. HC = healthy controls (*n* = 19), DN = DMARD naïve (*n* = 6), DR = DMARD responder (*n* = 24), D-NR = DMARD non-responder (*n* = 30), MFI = Median fluorescence intensity.

The frequency of CD43^+^ Tregs was reduced in each patient group compared to healthy controls (96.94% ± 2.434, *n* = 19), however only DMARD responders display a statistically significant difference (94.37% ± 2.698, *n* = 24, *p* = 0.001) (Figure 5). Furthermore, the frequency of CD43^+^ Tregs is significantly reduced in DMARD responders compared to DMARD naïve (96.37% ± 2.524, *n* = 6, *p* = 0.049) and non-responder patients (95.41% ± 4.647, *n* = 32, *p* = 0.024). Additionally, the MFI of CD43 on the surface of Tregs is reduced in each RA patient group compared to healthy controls (23.44MFI ± 27.96), however a statistically significant difference was only observed in non-responders (10.34MFI ± 11.55, *p* ≤ 0.001).

**Figure 6:**
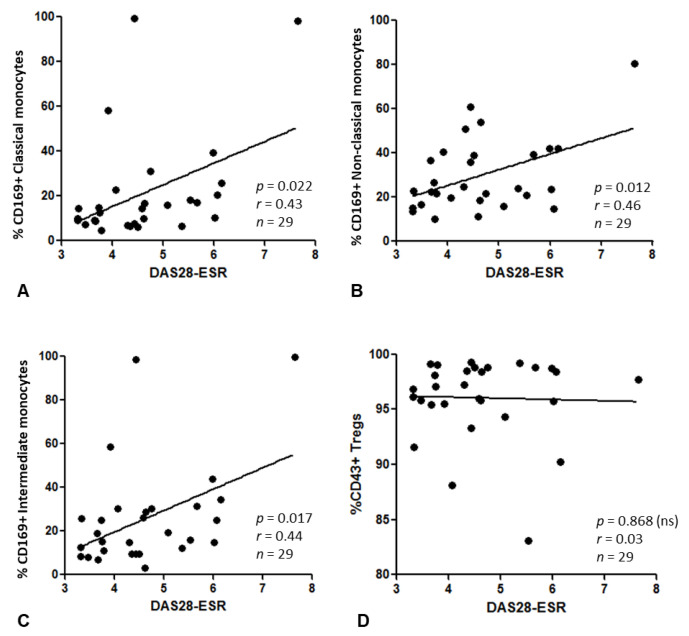
(**A**–**C**) The association between the frequency (%) of CD169^+^ classical (**A**), non-classical (**B**) and intermediate (**C**) monocytes (calculated as a % of CD14^+^ monocytes) and DAS28-ESR. (**D**) The association between CD43^+^ Tregs and DAS28-ESR. Linear regression was used to assess significance, with 95% confidence limits; ns, not significant. Data points show patients with moderate or high disease activity, as defined by EULAR criteria (*n* = 29).

A statistically significant positive association was observed between DAS28-ESR and the frequency of CD169^+^ classical (*p* = 0.022, r = 0.424, *n* = 29), non-classical (*p* = 0.012, r = 0.459, *n* = 29) and intermediate (*p* = 0.017, r = 0.440, *n* = 29) monocytes (Figure 6A–C). However, the frequency of CD169^+^ non-classical monocytes had the most significant relationship with DAS28-ESR versus other subgroups. The frequency of CD43^+^ Tregs had no significant association with DAS28-ESR (*p* = 0.868, r = 0.032, *n* = 29) (Figure 6D).

### 3.7. Effect of Sialic Acid on PMA Stimulated Tregs

**Figure 7:**
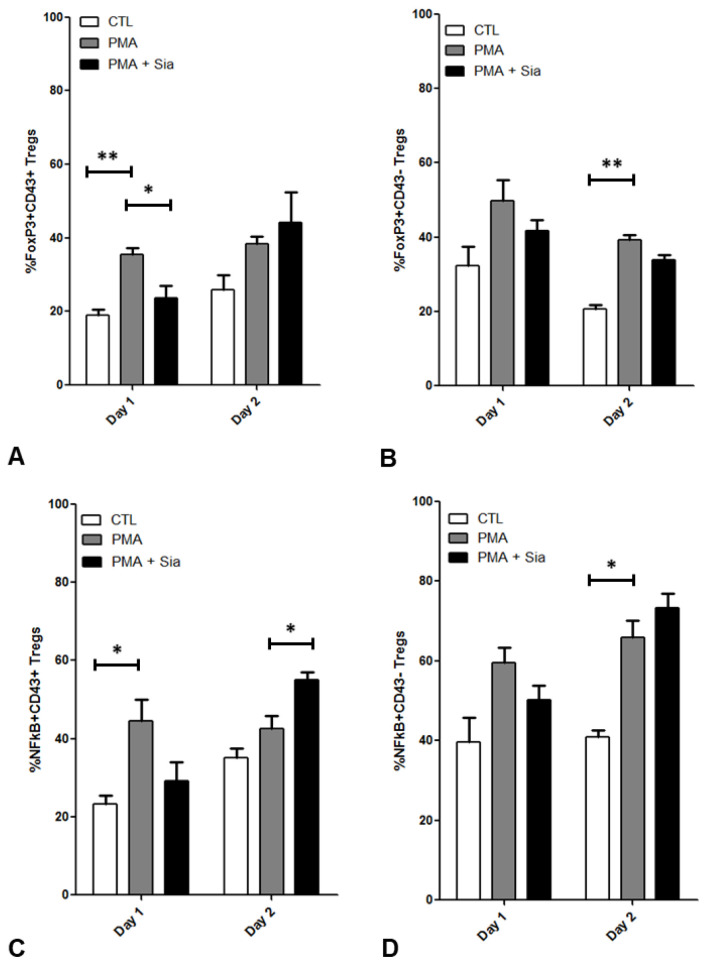
Flow cytometry data showing the change in relative frequency (%) of FoxP3^+^ and NFκB^+^ Tregs (CD4^+^CD25^+^CD127^−^), specifically (**A**,**C**) CD43^+^ and (**B**,**D**) CD43^−^ Treg cells following expansion in vitro for 14 days. Expanded Tregs were stimulated with 10 ng/mL PMA and/or 10 mM Sia for 24 h. Number of cell culture plate wells at each time point = 2 (mean ± SD plotted), where each was labelled for FACS analysis, and the experiment was repeated using a second donor. CTL = Control, PMA = Phorbol 12-myristate 13-acetate, Sia = Sialic acid. Unpaired t-tests were used to assess significance; *, *p* < 0.05; **, *p* < 0.01.

After stimulation with PMA, a reduction in CD4 cell-surface expression was observed (Supplementary Figure S1). Additionally, the percentage of CD43^+^ Tregs was significantly lower following in vitro expansion, compared to that observed from fresh PBMCs analysed on the day of collection. The percentage frequencies of NFκB and FoxP3 expressing cells were assessed in CD43^+^ (Figure 7A,C) and CD43^−^ Tregs (Figure 7B,D). PMA stimulation increased the percentage of cells with intracellular expression of NFκB (*p* < 0.05) and FoxP3 (*p* < 0.01) in both CD43^+^ and CD43^−^ Tregs at each time point. Addition of Sia reduced the relative numbers of FoxP3^+^ Tregs on Day 1 and Day 2 (*p* < 0.05). The same effect is observed after 2 days in CD43^−^FoxP3^+^ Tregs but not in CD43^+^FoxP3^+^ Tregs. Furthermore, after 2 days, the addition of Sia had no significant effect on NFκB^+^ Treg frequency.

**Figure 8:**
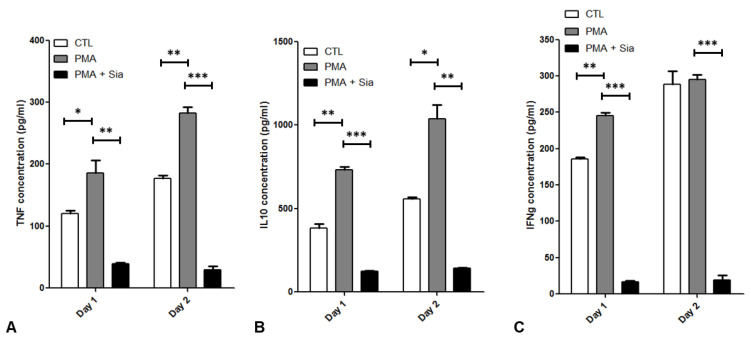
ELISA data assessing secreted cytokine levels from cell culture supernatants of CD4^+^CD25^+^CD127^−^ Tregs, expanded as described for 14 days in vitro. Expanded Tregs were stimulated with 10 ng/mL PMA and/or 10 mM Sia for 24 h. (**A**) TNFα, (**B**) IL-10 and (**C**) IFNγ levels were assessed 1 day and 2 days after treatment. Number of cell culture plate wells at each time point = 2, where each sample was run in duplicate wells of the ELISA plate (mean ± SD plotted), and the experiment was repeated using a second donor. CTL = Control, PMA = Phorbol 12-myristate 13-acetate, Sia = Sialic acid. T-tests were used to assess significance; *, *p* < 0.05; **, *p* < 0.01; ***, *p* < 0.001.

Concentrations of cytokines secreted into the surrounding media including TNFα, IL-10 and IFNγ were analysed by ELISA (Figure 8A–C). PMA significantly increased levels of TNFα (*p* < 0.05) and IL-10 (*p* < 0.05) on both Day 1 and Day 2, relative to controls. IFNγ levels were also significantly higher with PMA stimulation relative to controls on Day 1 (*p* < 0.01), but not on Day 2. At each time point, addition of Sia caused a significant reduction in TNFα, IL-10 and IFNγ (*p* < 0.01).

## 4. Discussions

This study provides novel insight into how the number and phenotype of peripheral Tregs relate to disease activity after DMARD treatment response in RA patients. Furthermore, novel findings supporting a potential role for monocyte involvement in modulation of Treg activity are presented, with particular focus on the interaction between CD169^+^ monocytes and CD43^+^ Tregs.

This study indicates that DMARD responder patients have an increased frequency (percentage) of Tregs compared to healthy controls, however DMARD non-responders exhibit reduced Treg numbers compared to responders. This is consistent with prior studies, whereby the frequency increases during anti-TNF response in naïve RA patients [9]. This may explain the significantly lower percentage of Tregs in DMARD non-responders observed in the current study, compared to responders. Given the modest number of DMARD naïve patients, the current study did not ascertain a statistically significant difference in relative Treg numbers, compared to DMARD responders.

CD45RA^+^ Tregs were also analysed, representing a naïve subset of Tregs that are not activated by prior antigen encounter. Within the CD45RA^+^ Tregs, the mean frequency of FoxP3^−^ cells is highest in DMARD non-responders compared to all of the other study groups. Furthermore, the frequency of FoxP3^+^ cells is reduced in DMARD responders and non-responders compared to healthy controls. This agrees with previous findings that activation of naïve Tregs is reduced in RA patients [34], however the difference observed has not been previously reported between responder and non-responder subgroups. Interestingly, the frequency of CD45RA^−^FoxP3^+^ Tregs was elevated in RA patients compared to healthy controls, in agreement with previous work [35]. This data suggests there is increased activation of CD45RA^−^ memory Tregs in RA peripheral blood, relative to healthy subjects. However, a significantly lower frequency of CD45RA^−^FoxP3^+^ Tregs is observed in DMARD non-responders compared to DMARD responder patients. This novel finding suggests DMARD non-responder RA patients exhibit reduced numbers of circulating active naïve and memory Tregs, than responders.

Increased absolute monocyte numbers were observed from full blood count (FBC) results of RA patients compared to healthy controls, which confirms that inflammatory recruitment of circulating monocytes can be detected. Similar to Chara et al., absolute numbers of monocytes are significantly reduced in DMARD responders compared to non-responders [29]. A further study by Chara et al., outlines how circulating monocytes increase in number during active disease [28]. This corroborates the higher monocyte numbers observed in RA patients that remain unresponsive to DMARDs, who have significantly higher disease activity.

In concordance with previous studies, the relative number of CD16^+^ monocytes was increased in RA [25,26], whereas CD16^−^ classical monocytes were reduced [27]. A previous study also found an increase in CD16^+^ intermediate monocytes in patients with longer disease duration compared to those with less than 2 years duration [36], however similar changes in CD16^+^ non-classical monocytes have not been previously reported. CD16^+^ monocytes, which are more likely to differentiate into dendritic cells [37], are believed to play a pivotal role in RA progression and could represent a novel therapeutic target [38]. However, despite evidence that classical and intermediate monocytes may predict treatment response [28], the current data indicates no significant difference in monocyte subsets between responders and non-responders.

The frequency of CD169^+^ classical, non-classical and intermediate monocytes was increased in RA compared to healthy controls, similar to findings by Xiong et al. [17]. However, a novel finding of the current study is that the frequency of CD169^+^ non-classical monocytes were significantly increased in RA compared to health. This result adds further weight to the idea that CD169^+^CD16^+^ monocytes may have a significant role in RA pathogenesis compared to CD16^−^ monocytes [17,37].

The cell surface density of CD43 (MFI) on circulating Tregs, as well as the frequency of CD43^+^ Tregs, is lower in RA compared to health which has not been reported previously. There is limited knowledge regarding the role of CD43 as a Treg transmembrane sialoglycoprotein, however previous studies have demonstrated its involvement in T cell activation and proliferation [39,40]. Therefore, the reduction in CD43^+^ cell absolute number and frequency observed in this study may be related to reduced Treg activation [11]. Contrary to this theory however, DMARD responders have significantly lower relative numbers of CD43^+^ Tregs compared to non-responders. Conversely the MFI of CD43 is increased in responders compared to non-responders, though the difference is not statistically significant. This means that although there may be a lower frequency of CD43^+^ Tregs in responders compared to non-responders, there is increased CD43 cell surface density per Treg cell in responders. Therefore, the increase in CD43 per cell in responders may be related to previously described increases in T cell activation [39,40], however further investigation is needed to confirm this. Although the CD43 profile may be difficult to interpret in this preliminary analysis, the difference in frequency between responders and non-responders would suggest CD43 has potential as a surrogate marker of treatment response.

This is the first immunophenotype study to report on the relationship between monocyte subsets and disease activity in RA. When monocyte subset was considered, the frequency of CD169^+^ monocytes had a significant association with DAS28-ESR. Interestingly, CD169^+^ non-classical monocytes had the strongest association with DAS28-ESR, whereas CD169^+^ classical monocytes had the weakest of the three subsets. This implies CD16^+^ monocytes are more closely associated with disease activity than CD16^−^ monocytes. There was no association between the frequency of CD43^+^ Tregs and DAS28-ESR, suggesting any impact of the CD169/CD43 relationship on disease activity may be dominated by CD169.

The aim of the in vitro inflammatory model was to induce protein kinase C pathway signaling in Tregs similar to activation observed in RA, as measured by production of pro-inflammatory cytokines including TNFα and IL-6. Sia was added to the activated Tregs to mimic the effect of CD169, which can bind to and present this residue. CD169 binds to Sia to carry out its functions, including binding to ligands on the surface of Tregs [20]. Although the purpose of the experiment was to determine cytokine and intracellular pathway responses, it cannot be definitively stated that CD169 has the same effect. Sia significantly reduced the activation of Tregs, as measured by decreased levels of secreted cytokines TNFα, IL-10 and IFNγ, as well as decreased relative numbers of NFκB^+^ and FoxP3^+^ Tregs. This agrees with previous studies, where Sia has been shown to reduce Treg suppressive function as well as activation markers including CD25 [20,41]. However, the impact on cytokine and transcription factors observed in the current study has not been previously reported. This effect was only observed in NFκB^+^ Tregs when they were activated with PMA/IO for 1 day. A similar effect was observed for NFκB^+^CD43^+^ and NFκB^+^CD43^−^ Tregs. However, after 2 days of stimulation Sia significantly reduced FoxP3^+^CD43^−^ Tregs, an effect which was not observed in FoxP3^+^CD43^+^ Tregs. The discrepancy between the frequency of NFκB^+^ and FoxP3^+^ Tregs, and cytokine levels on Day 2 of culture may occur as a result of an initial cytokine response within the first day of stimulation that is still detected in surrounding medium on Day 2.

Introducing Sia into PMA stimulated Treg cultures also significantly reduced TNFα, IL-10 and IFNγ cytokine secretion, an effect that was observed on both Day 1 and Day 2 of culture. We postulate that Sia may mimic the action of CD169 on Tregs, however this could not be confirmed with an anti-CD43 antibody. It could be postulated that direct interaction of CD169 and CD43 could contribute to Treg modulation and ultimately disease activity in RA, though further work is needed to confirm this.

In summary, this study presents novel evidence of peripheral Treg subsets in RA and an association between specific monocyte subsets and disease activity. Furthermore, this study suggests there is potential to assess disease activity by analysing circulating immune cells, which could enable earlier determination of treatment response.

## Figures and Tables

**Table 1:** Demographics of patients treated with conventional disease-modifying anti-rheumatic drugs, taken from clinic notes of the day of sampling. ESR = Erythrocyte Sedimentation Rate, CRP = C-reactive Protein, DAS28 = Disease Activity Score in 28 joints, HC = healthy control, DN = DMARD naïve, DR = DMARD responder, DNR = DMARD non-responder. Data is expressed as mean values and standard deviation (SD).

	HC (*n* = 21)	DN (*n* = 6)	DR (*n* = 24)	DNR (*n* = 32)
**Female, *n* (%)**	16 (76.2)	5 (83.3)	22 (91.7)	27 (81.8)
**Age, mean (SD), years**	35.9 (9.2)	42.5 (16.7)	59.7 (12.2)	57.7 (10.1)
**Disease duration, mean (SD), years**	N/A	N/A	5.0 (3.8)	10.8 (12.1)
**Lymphocytes, mean (SD), 10^9^/L**	1.9 (0.4), *n* = 19	1.6 (0.5), *n* = 6	1.7 (0.3), *n* = 24	1.6 (0.5), *n* = 26
**Monocytes, mean (SD), 10^9^/L**	0.5 (0.3), *n* = 19	0.6 (0.2), *n* = 6	0.5 (0.1), *n* = 24	0.6 (0.3), *n* = 26
**ESR, mean (SD), mm/h**	N/A	23.5, (15.2), *n* = 6	12.2 (7.9), *n* = 24	24.1 (20.0), *n* = 29
**CRP, mean (SD), mg/L**	N/A	6.1 (2.9), *n* = 6	5.3 (7.3), *n* = 22	10.6 (9.4), *n* = 28
**DAS28-ESR, mean (SD)**	N/A	4.3 (0.4), *n* = 4	2.6 (1.0), *n* = 24	4.9 (1.1), *n* = 19

**Table 2:** Statistical differences between clinical data of DMARD responders (DR) vs. DMARD non-responders (DNR). *p* values obtained using Mann–Whitney tests; *, *p* < 0.05; **, *p* < 0.01; ns, not significant. Data is expressed as median values and standard deviation (SD). ESR = Erythrocyte Sedimentation Rate, CRP = C-reactive Protein, DAS28 = Disease Activity Score in 28 joints.

	DR	DNR	*p* Value
**Disease Duration, years**	4.50 ± 3.85	9.00 ± 11.43	<0.01 (**)
**DAS28-ESR**	2.34 ± 1.05	5.51 ± 1.39	<0.01 (**)
**ESR,** mm/h	11.00 ± 7.93	21.50 ± 16.10	0.01 (*)
**CRP,** mg/L	2.75 ± 7.30	8.80 ± 24.80	0.08 (ns)

## Data Availability

The datasets used and/or analysed during the current study are available from the corresponding author on reasonable request.

## Supplementary Materials

The following supporting information can be downloaded at: https://www.mdpi.com/article/10.3390/jpm12111875/s1, Figure S1: (A) The frequency of CD43^+^ Tregs (Q2-1) was reduced after being placed in culture for 14 days in comparison to PBMCs analysed on the day of sampling. (B) (ii) The percentage of CD4 positivity (Q2) was reduced following PMA stimulation, compared to (i) CTL cells without stimulation.
