# Supplementary material for: CD169+ Monocyte and Regulatory T Cell Subsets Are Associated with Disease Activity in Rheumatoid Arthritis
Source: J Pers Med. 2022 Nov 9;12(11):1875. doi: 10.3390/jpm12111875 (PMC9695519; doi:10.3390/jpm12111875)
Supplement: Supplementary file 1 [file jpm-12-01875-s001.zip › jpm-1966809-supplementary.pdf]

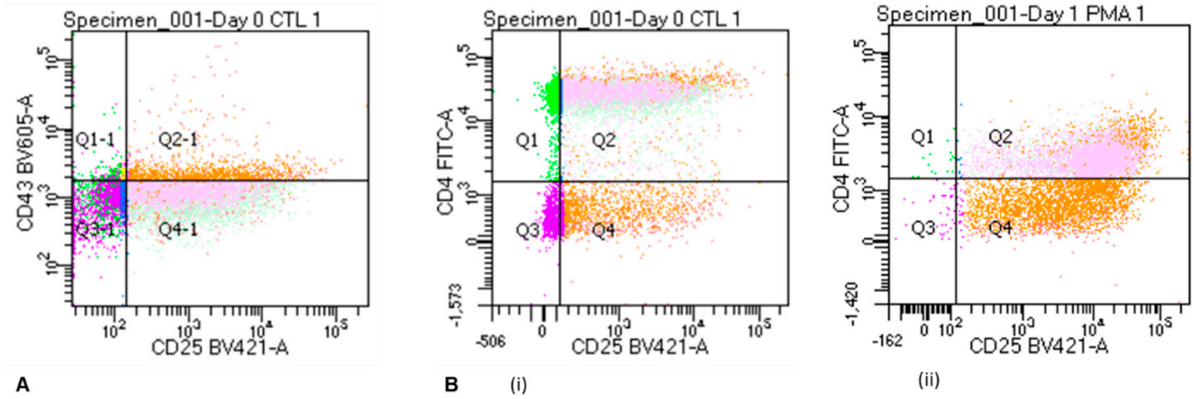


**Supplementary Figure S1.** (A) The frequency of CD43$^{+}$ Tregs (Q2-1) was reduced after being placed in culture for 14 days in comparison to PBMCs analysed on the day of sampling. (B) (ii) The percentage of CD4 positivity (Q2) was reduced following PMA stimulation, compared to (i) CTL cells without stimulation.
